# dbPTM in 2022: an updated database for exploring regulatory networks and functional associations of protein post-translational modifications

**DOI:** 10.1093/nar/gkab1017

**Published:** 2021-11-12

**Authors:** Zhongyan Li, Shangfu Li, Mengqi Luo, Jhih-Hua Jhong, Wenshuo Li, Lantian Yao, Yuxuan Pang, Zhuo Wang, Rulan Wang, Renfei Ma, Jinhan Yu, Yuqi Huang, Xiaoning Zhu, Qifan Cheng, Hexiang Feng, Jiahong Zhang, Chunxuan Wang, Justin Bo-Kai Hsu, Wen-Chi Chang, Feng-Xiang Wei, Hsien-Da Huang, Tzong-Yi Lee

**Affiliations:** The Genetics Laboratory, Longgang District Maternity & Child Healthcare Hospital of Shenzhen City, Shenzhen 518172, China; School of Life and Health Sciences, The Chinese University of Hong Kong, Shenzhen 518172, China; Warshel Institute for Computational Biology, The Chinese University of Hong Kong, Shenzhen 518172, China; Warshel Institute for Computational Biology, The Chinese University of Hong Kong, Shenzhen 518172, China; Warshel Institute for Computational Biology, The Chinese University of Hong Kong, Shenzhen 518172, China; Warshel Institute for Computational Biology, The Chinese University of Hong Kong, Shenzhen 518172, China; Warshel Institute for Computational Biology, The Chinese University of Hong Kong, Shenzhen 518172, China; School of Science and Engineering, The Chinese University of Hong Kong, Shenzhen 518172, China; Warshel Institute for Computational Biology, The Chinese University of Hong Kong, Shenzhen 518172, China; School of Science and Engineering, The Chinese University of Hong Kong, Shenzhen 518172, China; Warshel Institute for Computational Biology, The Chinese University of Hong Kong, Shenzhen 518172, China; School of Science and Engineering, The Chinese University of Hong Kong, Shenzhen 518172, China; Warshel Institute for Computational Biology, The Chinese University of Hong Kong, Shenzhen 518172, China; Warshel Institute for Computational Biology, The Chinese University of Hong Kong, Shenzhen 518172, China; School of Science and Engineering, The Chinese University of Hong Kong, Shenzhen 518172, China; Warshel Institute for Computational Biology, The Chinese University of Hong Kong, Shenzhen 518172, China; Warshel Institute for Computational Biology, The Chinese University of Hong Kong, Shenzhen 518172, China; School of Life and Health Sciences, The Chinese University of Hong Kong, Shenzhen 518172, China; Warshel Institute for Computational Biology, The Chinese University of Hong Kong, Shenzhen 518172, China; School of Life and Health Sciences, The Chinese University of Hong Kong, Shenzhen 518172, China; Warshel Institute for Computational Biology, The Chinese University of Hong Kong, Shenzhen 518172, China; School of Life and Health Sciences, The Chinese University of Hong Kong, Shenzhen 518172, China; Warshel Institute for Computational Biology, The Chinese University of Hong Kong, Shenzhen 518172, China; School of Life and Health Sciences, The Chinese University of Hong Kong, Shenzhen 518172, China; Warshel Institute for Computational Biology, The Chinese University of Hong Kong, Shenzhen 518172, China; School of Life and Health Sciences, The Chinese University of Hong Kong, Shenzhen 518172, China; Warshel Institute for Computational Biology, The Chinese University of Hong Kong, Shenzhen 518172, China; School of Life and Health Sciences, The Chinese University of Hong Kong, Shenzhen 518172, China; Warshel Institute for Computational Biology, The Chinese University of Hong Kong, Shenzhen 518172, China; Department of Medical Research, Taipei Medical University Hospital, Taipei 110, Taiwan; Institute of Tropical Plant Sciences and Microbiology, National Cheng Kung University, Tainan 701, Taiwan; The Genetics Laboratory, Longgang District Maternity & Child Healthcare Hospital of Shenzhen City, Shenzhen 518172, China; Department of Cell Biology, Jiamusi University, Jiamusi 154007, China; Shenzhen Children's Hospital of China Medical University, Shenzhen 518172, China; The Genetics Laboratory, Longgang District Maternity & Child Healthcare Hospital of Shenzhen City, Shenzhen 518172, China; School of Life and Health Sciences, The Chinese University of Hong Kong, Shenzhen 518172, China; Warshel Institute for Computational Biology, The Chinese University of Hong Kong, Shenzhen 518172, China; School of Life and Health Sciences, The Chinese University of Hong Kong, Shenzhen 518172, China; Warshel Institute for Computational Biology, The Chinese University of Hong Kong, Shenzhen 518172, China

## Abstract

Protein post-translational modifications (PTMs) play an important role in different cellular processes. In view of the importance of PTMs in cellular functions and the massive data accumulated by the rapid development of mass spectrometry (MS)-based proteomics, this paper presents an update of dbPTM with over 2 777 000 PTM substrate sites obtained from existing databases and manual curation of literature, of which more than 2 235 000 entries are experimentally verified. This update has manually curated over 42 new modification types that were not included in the previous version. Due to the increasing number of studies on the mechanism of PTMs in the past few years, a great deal of upstream regulatory proteins of PTM substrate sites have been revealed. The updated dbPTM thus collates regulatory information from databases and literature, and merges them into a protein-protein interaction network. To enhance the understanding of the association between PTMs and molecular functions/cellular processes, the functional annotations of PTMs are curated and integrated into the database. In addition, the existing PTM-related resources, including annotation databases and prediction tools are also renewed. Overall, in this update, we would like to provide users with the most abundant data and comprehensive annotations on PTMs of proteins. The updated dbPTM is now freely accessible at https://awi.cuhk.edu.cn/dbPTM/.

## INTRODUCTION

Post-translational modification (PTM) refers to the covalent processing of the translated proteins. PTMs modify specific amino acid residues to protein phosphorylation, glycosylation, ubiquitination, *S*-nitrosylation, methylation, acetylation, lipidation, and other modifications of proteins (1–3). PTMs are widely involved in the regulation of protein activity and function in organisms. Various types of modification greatly expand the chemical structure and functions of proteins, such as the spatial conformation and active state (4), subcellular localization (5), folding and stability (6,7), and protein–protein interactions (PPI) (8). These changes in physiochemical properties have significantly increased the diversity and complexity of proteins (9). Many vital life processes are controlled by the relative abundance of proteins, and importantly, regulated by PTMs (10,11). These processes include cell differentiation, protein degradation, signal transduction and regulatory processes, gene expression regulation and protein interactions (12–14). PTMs have been confirmed to be closely related to the occurrence and development of diseases such as heart disease, cancer, neurodegenerative diseases, and diabetes (15). Therefore, the characteristics of PTMs provide invaluable insights into the cellular functions under the etiological process. The study of PTMs may help clarify and understand the structure and function of proteins, which is also an important research content in proteomics and bioinformatics (16–19).

In recent years, the rapid development of mass spectrometry (MS)-based proteomics technology has greatly advanced the research progress of protein PTMs. Various new techniques for sample preparation, instrumentation, and MS data analysis have been developed and applied to PTM studies. New enrichment methods were used to identify some special PTM types. For example, mannose-6-phosphate glycosylation of proteins was enriched by dual-functional titanium (IV) immobilized metal affinity chromatography [Ti(IV)-IMAC] material (20). A highly specific pan-anti-Kla antibody was used to detect lactated modifications of histone lysines (21). Multiplexed isobaric labeling methods, such as 11-plex tandem mass tag (TMT), have been widely used for quantifying proteome and protein modifications (22). Recently, the technique has been updated to 16-plex TMT and utilized for proteomic profiling of biological and clinical samples (23). The development of MS technology also promotes the progress of PTM studies. High-field Asymmetric Waveform Ion Mobility Spectrometry (FAIMS) was introduced to improve the quantitative accuracy of PTM analysis (24,25). Sequential window acquisition of all theoretical fragment ion spectra (SWATH) presents great potential for target-free accurate identification and quantification of PTMs (26). The Data-Independent Acquisition (DIA) workflow is an MS data acquisition method that offers superior run-to-run consistency and post-acquisition flexibility in comparison to the Data-Dependent Acquisition (DDA) method (27). The application of these new techniques generates a large amount of raw data that requires proper tools for data analysis. Therefore, some data processing software has been invented to meet these requirements. For example, DIA-NN was developed to process the data generated by DIA-based proteomics experiments (28). The pFind3 could efficiently identify peptides with unexpected modifications, amino acid mutations, semi-specific or non-specific digestion and co-eluting peptides (29). MSFragger-Glyco is a search engine for fast and sensitive identification of N- and O-linked glycopeptides (30). New methods and techniques (31–33) have greatly facilitated the discovery of new types and sites of PTMs, which accumulated a large number of PTM sites.

The continuous discovery of new PTM sites has also stimulated people's interest in the mechanisms of PTM occurrence and their function in cells. Many studies have focused on the events that mediate the occurrence of PTMs, the operating mechanism of PTMs in cellular regulatory networks, and the effects of PTMs on cellular functions. These popular research topics have produced many meaningful findings. For example, histone acetyltransferase 1 (HAT1), a type B histone acetyltransferase, that succinylated histone H3 on K122, has been reported to contribute to epigenetic regulation and gene expression in cancer cells (34). Histone acetylation is a crucial PTM type that contributes to tumorigenesis by promoting the expression of YTHDF2, which is associated with poor prognosis in ocular melanoma (35). PTMs are also associated with cellular metabolic alteration. Changes in lysine acetylation in key enzymes may impair their activities and alter the metabolic homeostasis of the follicular microenvironment of oocyte maturation and embryonic development (36). Enhancement of glutaminase (GLS) K311 succinylation may promote tumor cell survival and tumor growth by increasing glutaminolysis and the production of nicotinamide adenine dinucleotide phosphate (NADPH) and glutathione through counteracting the oxidative stress (37). The crosstalk between acetylation and ubiquitination in AMPA receptors (AMPARs) presents crucial roles in synaptic plasticity and memory (38). *Toxoplasma. gondii* (T. gondii) infection inhibits the crotonylation of H2B on K12, which suppresses the epigenetic regulation and NF-κB activation, and provides a basis for studying the immune response mechanism of host cells against T. gondii infection (39). These new findings expand our understanding of PTMs. The analysis of PTMs has important implications for understanding the basic biological processes and the occurrence of diseases.

Hence, to facilitate the study of protein PTMs, dbPTM (40) has been developed as a comprehensive database that provides functional and structural analyses for PTM sites. This update accumulates more than 2 777 000 PTM substrate sites from existing databases and manually curated literature, of which over 2 235 000 entries are experimentally verified. In this updated version, 76 PTM types are curated, 42 of which were not previously covered. A total of 44 753 relationships between the upstream regulatory proteins and PTM substrate sites were embedded in the updated dbPTM and integrated into the PPI network. Functional annotations of PTMs were collected using text mining and manual auditing to deepen the understanding of the association between PTMs and molecular functional/physiological processes. In addition, new online databases and tools related to PTM analysis were organized and integrated into the existing PTM analysis resource portal. Overall, with this update, we expect dbPTM to become a one-stop database and service platform for PTM studies. It will provide users with valuable resources of protein PTMs and promote a deeper understanding of PTM functions and regulatory mechanisms.

## DATA COLLECTION AND PROCESSING

### Integration of site-specific PTMs

The dbPTM has integrated comprehensive PTM sites from public biological databases, including UniProtKB/Swiss-Prot (41), PhosphoSitePlus (42), ActiveDriverDB (43), etc. In addition, PTM-related articles were systematically retrieved by query of PTM-related keywords, such as phosphorylation, ubiquitination, or acetylation, in the fields ‘Title’ and ‘Abstract’ from PubMed. Then, the obtained articles were manually reviewed to extract MS/MS verified PTM sites along with the corresponding sequences of substrate residues. Up to 310 research articles related to protein PTMs were retrieved from PubMed (starting from Jan. 2019). These articles were manually reviewed one by one. The PTM sites identified by MS/MS in the articles were extracted so that each PTM site had a corresponding PubMed ID. To solve the heterogeneity among data collected from different sources, the obtained PTM sites were mapped to the UniProtKB protein entries using sequence comparison. Only the sites with the same protein sequence were retained. These PTM sites obtained from public resources and research articles were integrated with the previous version of dbPTM data to form a new dataset after de-redundancy.

### Upstream regulatory proteins of PTMs

Upstream proteins, such as kinases and E3 ligases, are the key to regulating the occurrence of PTMs. In this update, we compiled kinase-specific phosphorylation sites and E3 ligase-substrate interactions from UbiNet 2.0 (44), UniProtKB/Swiss-Prot (41), GPS 5.0 (45), PhosphoSitePlus (42) and other databases. The upstream regulatory relationships obtained from GPS 5.0 were all experimentally validated, and thereby used as the training dataset for model construction of kinase-specific phosphorylation sites. All redundant records were removed and the rest were integrated into the construction of the PTM regulatory network, which was primarily derived from BioGRID (46).

### Functional annotations associated with PTM substrate sites

All the records of reviewed proteins from five organisms, including human (20 395), mouse (17 073), *Arabidopsis thaliana* (16 043), rat (8125) and *Saccharomyces cerevisiae* (6721), were downloaded from UniProtKB/Swiss-Prot, along with the attribute of PTM description (41). The description was filtered to obtain 13 844 records with PTM functions descriptions. After that, a concise text-mining program was applied to the extraction of PTM function description sentences for each PTM site. First, texts of PTM functional descriptions were separated based on paragraph segmentation. Second, sentence tokenization is performed using Natural Language Toolkit (NLTK) (47). Third, after obtaining each sentence, a ‘regular matching’ function was used to detect sentences containing specific ‘PTM types’ and ‘PTM sites’. For instance, the sentence ‘Glycosylation at least at one of the two sites Asn-51 and Asn-301 is necessary for enzyme stability and activity.’, containing ‘Glycosylation’, ‘Asn’, ‘–’ and ‘301(number)’, was identified as a PTM functional description. Then, XML documents were crawled from UniProtKB/Swiss-Prot and parsed to map supporting references to corresponding functions of PTMs. Finally, all obtained records were manually checked to ensure that those sentences were functional descriptions of PTMs.

## DATABASE FEATURES AND APPLICATIONS

**Figure 1.**
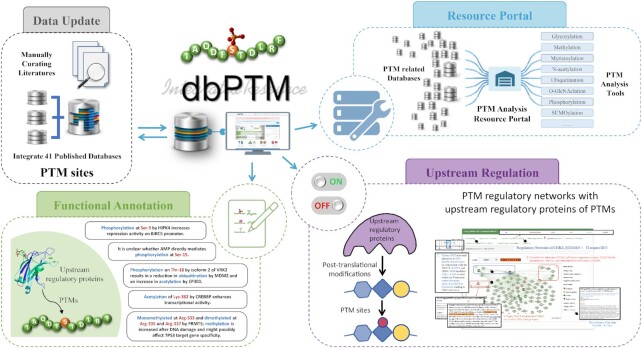
Schematic diagram of the improvements and advances in dbPTM 2022 update.

The highlighted improvements and advances in the dbPTM 2022 update are presented in Figure 1, such as updating site-specific PTMs from published databases and literature, the reconstruction of PTM regulatory networks using upstream regulatory proteins of PTMs, the integration of functional annotations associated with PTM substrate sites, and the update of existing PTM analysis resource portal.

### Database content and data statistics

In the updated dbPTM, a large amount of PTM sites were obtained from both external data sources and manual curation of the literature, resulting in 2 777 771 PTM sites, with an increase of more than 1 520 000 over the previous version. The number of PTM substrate species was enriched to 7070 (Supplementary Table S1), which is much greater than the previous version (1550). Among these 7070 species, 5922 have more than one annotated PTM, and 2898 species have more than 10 annotated PTMs. The data was comprehensively collected from 41 external databases (30 in the previous release). Of all databases, the dbPTM contains the largest number of PTM sites (including the experimental sites and the putative sites) and literature. The comparison of data statistics of PTM sites between dbPTM and these external databases was provided in Supplementary Table S2. There are 2 235 664 experimentally verified PTM sites in this updated version extracted from 82 444 articles, with an increase of more than 1 326 000 sites comparing to the previous version. The number of disease-associated PTM sites and the PTM pairs (PTM crosstalk) is increased. The number of disease-associated PTM sites based on disease-associated non-synonymous SNPs and Genome-Wide Association Studies (GWAS) increased from 350 to 2846, involving up to 30 PTM types. Protein phosphorylation has the most abundant data associated with disease traits, with up to 1892 substrate sites. These phosphorylation sites are closely associated with diseases such as coronary heart disease.

The increasing number of studies exploring the crosstalk between different PTMs had inspired us to design a platform for investigating the relative frequency and functional relevance of PTM co-occurrences on several modification types, fabricating the previous version. In this update, the investigation of PTM crosstalk between two different types is also a highlighted improvement. We found 370 PTM pairs that show cased co-occurrences of PTM sites, which are illustrated in summary tables on the web interface. If users are interested in a specified PTM type, a summary table is generated to provide all other PTM types co-occurring within a window length (–10 to +10 AA) to the specified PTM sites (centred at position 0). For example, if users search for the protein methylation (Supplementary Figure S1), as shown in the first column, users can review a total of 238 proteins containing the O-linked glycosylation sites co-occurring with the methylation sites in a specified window length. Among them, a total of 39 proteins consists of the O-linked glycosylation sites occurring at position 8 corresponding to the methylation sites at position 0. Additionally, users can access functional enrichment analysis results for two specified PTMs by clicking on the corresponding numbers in the first column which indicate the number of proteins with the specified PTM crosstalk. By clicking on the numbers at a certain position within the window length, users can browse the proteins that have corresponding PTM crosstalk at the specified distance (Supplementary Figure S1).

In addition to the PTM crosstalk analysis, 12 079 records of functional annotations of PTM sites and 44,753 upstream regulatory relationships are newly included in dbPTM, which were used for the reconstruction of PTM regulatory networks and the integration of functional annotations associated with PTM substrate sites. The comparison of data statistics and relevant information between this update and the previous version was displayed in Table 1. This version of dbPTM contains a total of 76 PTM types. Supplementary Table S3 shows the number of experimental, putative, and total substrate sites for each PTM type. Among all modification types, the number of protein phosphorylation sites is the largest, exceeding 63.9% of the total sites. The second enriched PTM type is ubiquitination, at approximately 16.4%. The number of PTM sites for these two PTM types has been greatly increased compared with the previous version, which indicates that protein phosphorylation and ubiquitination are the most popular research topics in the proteomics community in recent years.

**Table 1.** Comparison of data statistics of relevant information between dbPTM 2022 and the previous version.

Description	dbPTM 2019	dbPTM 2022
Experimental validated PTM sites	908 917	2 235 664
Species of PTM tdsubstrates	1550	7070
PTM types	34	76
Integrated online databases and tools	148	258
Integrated PTM resources	30	41
Disease-associated PTM sites	350	2846
PTM pairs (PTM crosstalk)	169	370
Functions of PTM sites	N/A	12 079
Upstream regulatory relationships	N/A	44 753

### Construction of PTM regulatory networks using upstream regulatory proteins

The occurrence of protein PTMs is exquisitely regulated by upstream regulatory proteins. Exploring the specific relationships between modifying substrates and their regulatory proteins is essential for understanding the functional role of PTMs. For example, kinases, one of the largest families of proteins in eukaryotes, regulate the mechanism of phosphorylation (48–50). Typically, a kinase recognizes one to a few hundred bona fide phosphorylation sites in nearly 700 000 potentially phosphorylatable residues (51). This regulatory relationship is essential for the phosphorylation cascade that causes a chain reaction leading to the phosphorylation of thousands of proteins. Another example is E3 ubiquitin ligases, which are important participants in the regulation of protein ubiquitination. In the process of protein ubiquitination, ubiquitin is attached to substrate proteins by a three-step process involving three enzymes: ubiquitin-activating enzyme (E1), ubiquitin-conjugating enzyme (E2), and ubiquitin–protein ligase (E3). Among them, E3 ubiquitin ligases determine the recognition of ubiquitinated substrates and thus play crucial roles in the ubiquitin-proteasome system. In fact, there are many other upstream regulatory enzymes that have similar roles in other types of PTMs. Therefore, the knowledge of upstream regulatory proteins of PTMs is the basis for studying the functional mechanism of PTMs.

**Figure 2.**
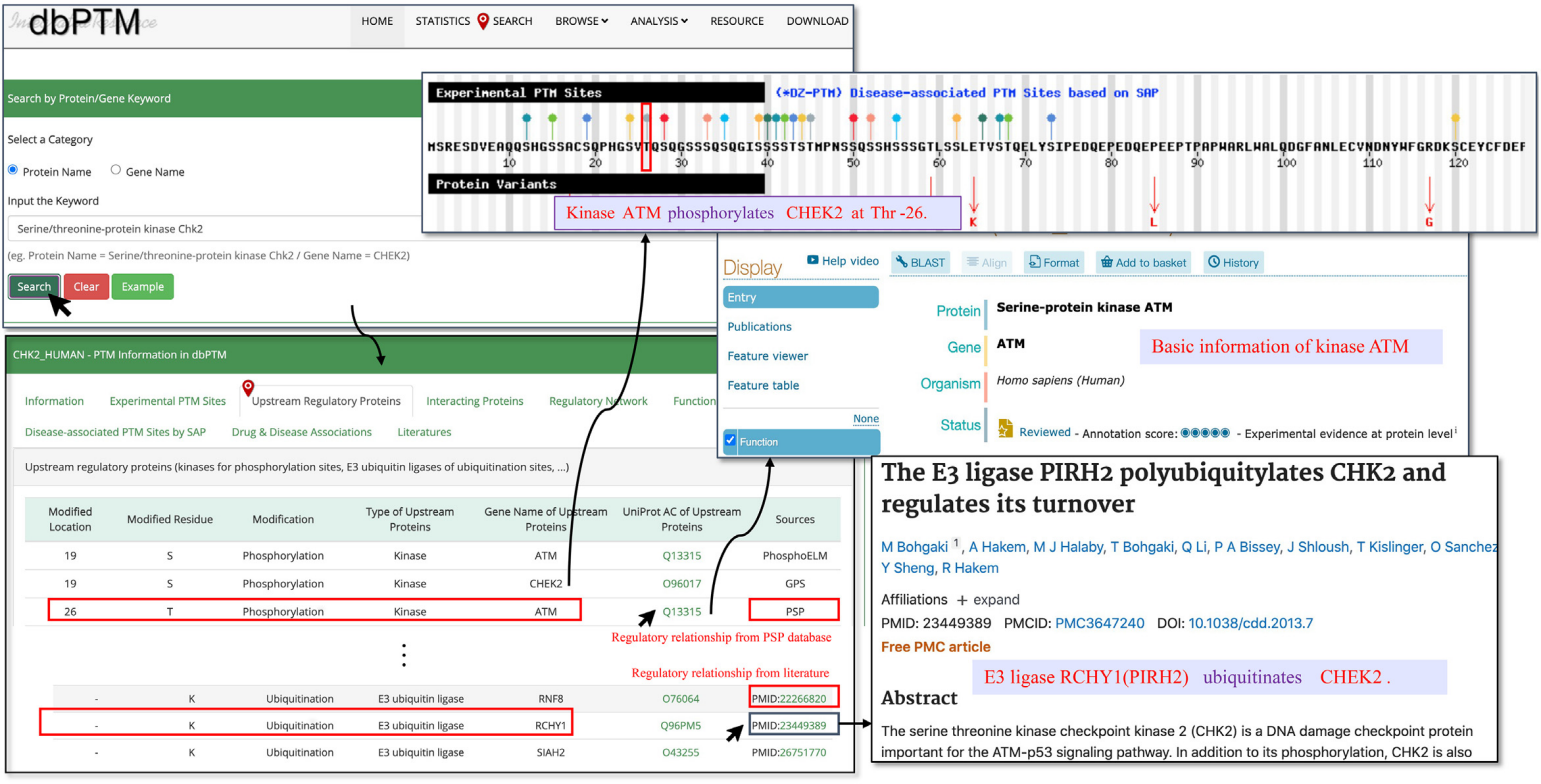
A case study to present the upstream regulatory proteins of serine/threonine–protein kinase Chk2. The known kinases that phosphorylate Chk2 for each site and E3 ubiquitin ligases modifying Chk2 are illustrated in the web page. The sources of each item including external databases and literatures are shown as well.

In this update, we compiled kinase-specific phosphorylation sites and E3 ligase-substrate interactions from UbiNet 2.0 (44), UniProtKB/Swiss-Prot (41), GPS 5.0 (45), PhosphoSitePlus (42) and other databases. After the removal of redundant entries, a total of 44 753 upstream regulatory relationships were embedded in the updated dbPTM (Supplementary Table S4). Figure 2 presents a case study on the upstream regulatory proteins of serine/threonine-protein kinase Chk2 (CHEK2). In the web interface of dbPTM, the ‘Upstream Regulatory Proteins’ provides a list of known kinases that phosphorylate Chk2 at each substrate site and the verified E3 ubiquitin ligases modifying Chk2 with the annotations of modified location, modified residue, modification type, type of upstream proteins, gene name of upstream proteins, UniProt AC of the upstream proteins and resources (databases or supporting references). Users can browse the upstream regulatory proteins of the queried protein using the ‘Search’ item in the navigation menu at the top of the web page.

**Figure 3.**
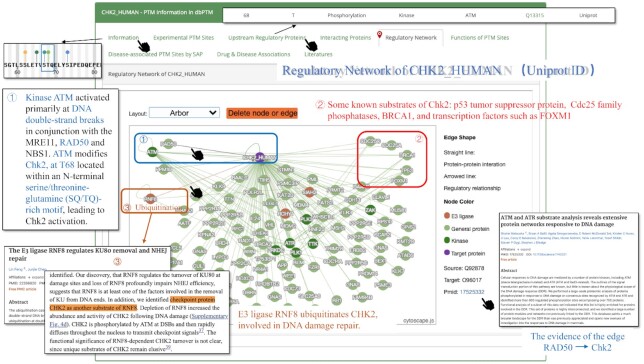
The regulatory network of CHK2. The colors of dots represent the different roles of the nodes. Purple: the target protein, CHK2 (the keyword searched: CHK2_HUMAN); brown: E3 ligase; green: kinase; and light green: general proteins.

To visualize the regulatory relationships, we integrated them into the PPI network, which can be used as a draft on which the regulatory pathways of a particular protein can be further investigated. Figure 3 shows a scheme for users to access the regulatory network of Chk2. By clicking on the edges, users can access the regulatory relationships and supporting references. Different types of proteins, including kinases, E3 ubiquitin ligases, substrates, and general proteins are indicated by different colors. As shown in Figure 3, Chk2 plays a key role in the DNA damage response (52). Kinase ATM is recruited and activated primarily at DNA double-strand breaks (DSBs) in conjunction with the RAD50, MRE11, and NBS1 sensor complex (53). Chk2 is then phosphorylated by ATM on Thr-68 within the N-terminal SQ/TQ-rich domain to trigger downstream responses (54). It is also reported that the substrates of Chk2 include the p53 tumor suppressor protein (55), MDMX (56), Cdc25 family phosphatases, tumor suppressor BRCA1 (57) and transcription factors FOXM1 (58). In addition, Lin *et al.* found that Chk2 is the substrate of E3 ligase RNF8. Depletion of RNF8 increased the abundance and activity of CHK2 following DNA damage (59). In short, this network analysis has demonstrated its capability of illustrating PTM and other regulatory information related to the target protein, and its efficiency in displaying the network to users in a structured way.

### Integration of regulatory functions of PTM sites in various cellular processes

**Figure 4.**
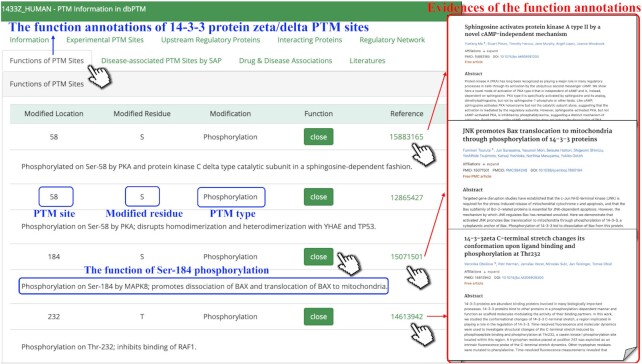
The functional annotations of PTM sites. The brief functional descriptions of 14–3–3 protein zeta/delta PTM sites are provided. The corresponding supporting references can be viewed by clicking on PubMed IDs.

PTMs that occur at specific sites are closely related to the physiological or pathological processes of cells. It is well known that PTMs at distinct sites usually have different functions. For example, as shown in Supplementary Figure S2, phosphorylation at Thr68 of serine/threonine-protein kinase Chk2 induces its homodimerization (60), while phosphorylation at Ser73 by PLK3 promotes its phosphorylation at Thr68 (61). Despite the importance of functions of PTMs at specific sites, few PTM databases provide functional descriptions of PTM sites, including the previously released dbPTM. This is a flaw that cannot be ignored because if users want to understand the functions of PTMs at different sites, they have to take time to check the references one by one. Therefore, we integrated the functions of PTMs at specific sites into this update. In total, we collected 12 079 records of human, mouse, A.thaliana, rat and *S. cerevisiae* by combining text mining and manual checking. It is worth mentioning that these text records are able to form a corpus to provide a dataset for future text mining-based model construction. Figure 4 shows an example of the functional annotations of each PTM site for 14–3–3 protein zeta/delta. Bax, a Bcl-2 family member, is crucial in inducing apoptosis in response to stress stimuli (62). Protein 14–3–3, the cytoplasmic anchor of Bax, prevents apoptosis by sequestration of Bax (63). It is reported that the phosphorylation at Ser184 of 14–3–3 promotes dissociation of Bax and translocation of Bax to mitochondria, leading to apoptosis (64). However, the phosphorylation at Ser58 of 14–3–3 regulates its dimeric status (65).

### Enhancement of the existing PTM analysis resource portal

The previous version of dbPTM established an extensive resource portal for PTM analysis, providing a convenient entrance for researchers to find appropriate databases or tools to analyze single or multiple PTMs of interest. With the rapid growth of PTM sites verified by MS/MS-based proteomics technology, many new online databases and tools for PTM analysis have been developed in recent years, such as PTM data warehouse, PTM site prediction tools, and regulatory network of PTMs with other proteins. In this update, we organized these newly developed resources to enrich the portal. The integrated PTM databases and tools in the resource portal, including their names and applicable PTM types, could be referred to Supplementary Table S5. Users can access these integrated databases and tools through this portal to easily and efficiently acquire resources related to PTM analysis. For example, users can browse a list of all relevant databases and tools according to the PTM type of their interests, without having to spend a lot of time and effort searching the Internet or literature databases. With the update of the resource portal, the dbPTM can provide users with effective access to all online resources related to PTMs. A tutorial for browsing online resources of interested PTM type was attached to Supplementary Figure S3.

### An easy-to-use and professional PTM analysis platform

The dbPTM provides a user-friendly interface for biologists to investigate PTMs in detail (Supplementary Figure S4). Users are allowed to browse PTM sites in three different ways according to their needs. Specifically, all of the PTM substrate sites in dbPTM can be browsed by specifying species, PTM types and modified residues. Users can also access disease-associated PTM sites and drug-associated PTM sites by selecting specific PTM types. As an aggregated PTM information platform, the general physical and chemical properties of the modified residues are listed in the ‘PTM general information’ section. In addition, a large number of PTM analysis resources are integrated and can be accessed via the ‘Resource’ item of the navigation menu at the top of the web page. Based on the collected substrate proteins, we performed PTM crosstalk analysis and functional enrichment analysis for each PTM pair. The results are provided and demonstrated graphically through the ‘Analysis’ item. Additionally, the ‘Search’ function allows users to efficiently reach the PTM data matching the querying criteria. For substrate proteins of interest, the dbPTM provides comprehensive available information about PTMs, including graphical visualization of PTM sites with structural characteristics and functional domains, a table of experimental PTM sites with supporting references, orthologous conservation of PTM substrate sites, disease-associated PTM sites, PPI and domain-domain interactions, a table of disease and drug associations as well as literature related to PTMs. It also provides three newly developed functions in this update including ‘Upstream regulatory proteins’, ‘Regulatory Network’ and ‘Functions of PTM sites’, leading to a one-stop PTM information and analytics platform.

## CONCLUSION

With the rapid development of MS-based proteomics research, the content of the dbPTM database is also continuously updated. Through manually curating the literature, recruiting curators and integrating databases and tools, the dbPTM 2022 has achieved outstanding improvements and advancements. So far, the dbPTM has curated 2 777 771 PTM sites from 41 published databases and 82 444 research articles. To help users conduct PTM analyses quickly and effectively, 73 databases and 185 tools related to more than 40 PTM types were collected to update the previously established resource portal. The PTM regulatory networks have been reconstructed by using 44 753 upstream regulatory relationships. In addition, the updated dbPTM has integrated 12 079 records of functional annotation associated with PTM sites. The network analysis and functional annotations would expand the understanding of the regulatory mechanism of PTMs and their functional roles in cells. In summary, with this update, we have integrated more PTM knowledge into dbPTM to create a comprehensive resource portal that can provide truly valuable contributions to PTM research community.

## DATA AVAILABILITY

The dbPTM will be maintained and updated quarterly by continuously surveying the public resources and research articles. The updated resource is now freely accessed online at https://awi.cuhk.edu.cn/dbPTM/. All the PTM sites could be downloaded in text format.

## Supplementary Material

gkab1017_Supplemental_Files
