# Supplementary material for: dbPTM in 2022: an updated database for exploring regulatory networks and functional associations of protein post-translational modifications
Source: Nucleic Acids Res. 2021 Nov 12;50(D1):D471–9. doi: 10.1093/nar/gkab1017 (PMC8728263; doi:10.1093/nar/gkab1017)
Supplement: gkab1017_Supplemental_Files [file gkab1017_supplemental_files.zip › Supplementary Figures S1-4 and Tables S2-5.docx]

**Supplementary Figures S1-S4 and Tables S2-S5**

**Figure S1.** A tutorial for using the summary table of PTM crosstalk between two different types and querying their functional enrichment analysis results.

**Figure S2.** The functional annotations of each PTM site. The functions of serine/threonine-protein kinase Chk2 PTM sites are briefly described.

**Figure S3**. A tutorial for browsing the online resources of interested PTM type. The resource portal has integrated online resources as fully as possible for PTM analysis, including databases and tools. Users could select a PTM type of interest and then a list of resource names, descriptions, website links, and corresponding references are provided to users. The portal allows users to browse easily and access efficiently to the appropriate resources by clicking on the website link to connect to them, which assists users with further PTM analysis in a more targeted and efficient way.

**Figure S4**. Schematic representation of the services provided in dbPTM 2022 update.

**Table S2. Comparison of data statistics of PTM sites between dbPTM and other PTM databases.**

| Resource | Version | Number of PTM sites | Number of experimental Sites | Number of putative sites | Number of Literatures |
| --- | --- | --- | --- | --- | --- |
| ProteomeScout | Release 2021_03 | 723,113 | 228,688 | 494,425 | 40 |
| SysPTM | Release 2.0 | 514,766 | 41,340 | 473,426 | 1,352 |
| dbPAF | Release 2021_05 | 483,001 | 483,001 | 0 | 15,139 |
| PhosphoSitePlus | Release 2018_05 | 428,476 | 85,136 | 343,340 | 79,935 |
| ActiveDriverDB | Release 2021_03 | 261,348 | 60,897 | 254,451 | 6,263 |
| UniProtKB | Release 2021_03 | 396,480 | 106,129 | 290,351 | 10,727 |
| CPLM | Release 2021_05 | 189,919 | 0 | 189,919 | 0 |
| PLMD | Release 3.0 | 184,779 | 184,417 | 362 | 1,590 |
| SubPhosDB | Release 2013_07 | 137,153 | 0 | 137,153 | 0 |
| mUbiSiDa | Release 2013_10 | 111,208 | 111,208 | 0 | 102 |
| HPRD | Release 9.0 | 93,710 | 49,811 | 43,899 | 1,832 |
| Ubisite | Release 1.0 | 92,432 | 91,588 | 844 | 24,988 |
| PHOSIDA | Release 3.69 | 80,062 | 80,062 | 0 | 17 |
| dbPPT | Release 1.0 | 69,297 | 69,297 | 0 | 79 |
| Phospho.ELM | Release 9.0 | 50,660 | 50,622 | 38 | 3,589 |
| UbiNet | Release 1.0 | 44,175 | 44,175 | 0 | 233 |
| PhosphoGRID | Release 2.0 | 42,908 | 42,908 | 0 | 528 |
| ESBL | Release 2018_05 | 20,001 | 9,970 | 10,031 | 5 |
| dbPSP | Release 2.0 | 19,296 | 19,296 | 0 | 471 |
| SwissPalm | Release 3.0 | 7,452 | 7,452 | 0 | 760 |
| ASEB | Release 2015_12 | 4,875 | 4,875 | 0 | 281 |
| CarbonylDB | Release 2018_02 | 4,210 | 4,210 | 0 | 115 |
| dbSNO | Release 2.0 | 4,165 | 4,161 | 4 | 175 |
| dbGSH | Release 1.0 | 4,119 | 4,119 | 0 | 60 |
| OGlycBase | Release 6.0 | 2,221 | 2,211 | 10 | 178 |
| ProteomeXchange | Release 2021_04 | 539 | 464 | 75 | 243 |
| Mal-Lys | Release 1.0 | 483 | 0 | 483 | 0 |
| MeMo | Release 2.0 | 364 | 364 | 0 | 57 |
| PupDB | Release 1.3 | 311 | 195 | 116 | 4 |
| PredGPI | Release 1.0 | 26 | 0 | 26 | 0 |
| The O-GlcNAc Database | Release 1.2 | 11,201 | 11,201 | 0 | 2,211 |
| Biogrid | Release 2021_04 | 553,360 | 553,360 | 0 | 4,653 |
| EPSD | Release 2019_11 | 1,616,804 | 1,616,804 | 0 | 38,574 |
| MetOSite | Release 2021_01 | 7588 | 7588 | 0 | 124 |
| DeepKhib | Release 1.0 | 39,970 | 39,970 | 0 | 24,815 |
| iHyd-PseCp | Release 2016 | 993 | 993 | 0 | 1 |
| HydLoc | Release 2020 | 645 | 645 | 0 | 1 |
| LipoPred | Release 2018 | 52 | 52 | 0 | 1 |
| NeddPred | Release 2019 | 51 | 51 | 0 | 1 |
| PredNTS | Release 2021 | 1,386 | 1,386 | 0 | 1 |
| OGP | Release 2021 | 9,348 | 9,348 | 0 | 1 |
| dbPTM | Release 2019 | 1,256,901 | 908,917 | 347,984 | 92,648 |
| dbPTM | Release 2022 | 2,777,771 | 2,235,664 | 542,107 | 82,444 |

**Table S3. The statistics of experimental and putative PTM sites in dbPTM**

| **PTM type** | **Number of experimental sites** | **Number of putative sites** | **Total sites of the type** | **Number of literatures** |
| --- | --- | --- | --- | --- |
| Acetylation | 138,171 | 38,530 | 176,701 | 19,451 |
| ADP-ribosylation | 107 | 499 | 606 | 35 |
| Amidation | 3,316 | 1,462 | 4,778 | 1,084 |
| AMPylation | 27 | 65 | 92 | 20 |
| Biotinylation | 12 | 83 | 95 | 13 |
| Blocked amino end | 26 | 73 | 99 | 26 |
| Butyrylation | 82 | 269 | 351 | 4 |
| Carbamidation | 22 | 0 | 22 | 1 |
| Carboxyethylation | 1 | 5 | 6 | 1 |
| Carboxylation | 44 | 1,513 | 1,557 | 63 |
| Cholesterol ester | 2 | 28 | 30 | 2 |
| Cholesterylation | 0 | 2 | 2 | 0 |
| Citrullination | 122 | 362 | 484 | 18 |
| C-linked Glycosylation | 211 | 149 | 360 | 19 |
| Crotonylation | 373 | 286 | 659 | 7 |
| Deamidation | 92 | 382 | 474 | 44 |
| Deamination | 42 | 150 | 192 | 14 |
| Decanoylation | 6 | 1 | 7 | 7 |
| Decarboxylation | 2 | 2 | 4 | 3 |
| D-glucuronoylation | 1 | 0 | 1 | 1 |
| Farnesylation | 86 | 355 | 441 | 83 |
| Formation of an isopeptide bond | 29 | 228 | 257 | 17 |
| Formylation | 256 | 51 | 307 | 47 |
| Gamma-carboxyglutamic acid | 469 | 717 | 1,186 | 97 |
| Geranylgeranylation | 81 | 1,051 | 1,132 | 51 |
| Glutarylation | 952 | 704 | 1,656 | 5 |
| Glutathionylation | 4,129 | 44 | 4,173 | 86 |
| Glycation | 0 | 323 | 323 | 0 |
| GPI-anchor | 103 | 1,021 | 1,124 | 57 |
| Hydroxyceramide ester | 3 | 1 | 4 | 1 |
| Hydroxylation | 2,404 | 4,543 | 6,947 | 353 |
| Iodination | 19 | 70 | 89 | 3 |
| Lactoylation | 306 | 587 | 893 | 2 |
| Lactylation | 336 | 0 | 336 | 2 |
| Lipoylation | 35 | 566 | 601 | 30 |
| Malonylation | 12,847 | 145 | 12,992 | 12 |
| Methylation | 16,114 | 16,766 | 32,880 | 8,897 |
| Myristoylation | 312 | 1,460 | 1,772 | 207 |
| N-carbamoylation | 1 | 0 | 1 | 1 |
| Neddylation | 1,676 | 48 | 1,724 | 4 |
| Nitration | 81 | 1,604 | 1,685 | 18 |
| N-linked Glycosylation | 27,366 | 89,143 | 116,509 | 1,952 |
| N-palmitoylation | 74 | 2,189 | 2,263 | 64 |
| Octanoylation | 7 | 6 | 13 | 8 |
| O-linked Glycosylation | 16,696 | 8,809 | 25,505 | 4,563 |
| O-palmitoleoylation | 6 | 188 | 194 | 11 |
| O-palmitoylation | 3 | 11 | 14 | 4 |
| Other | 1,257 | 42,535 | 43,792 | 344 |
| Oxidation | 427 | 774 | 1,201 | 84 |
| Phosphatidylethanolamine amidation | 9 | 83 | 92 | 7 |
| Phosphoglycerylation | 0 | 141 | 141 | 0 |
| Phosphorylation | 1,615,150 | 160,490 | 1,775,640 | 44,985 |
| Propionylation | 13 | 159 | 172 | 4 |
| Pupylation | 0 | 215 | 215 | 0 |
| Pyrrolidone carboxylic acid | 941 | 832 | 1,773 | 571 |
| Pyrrolylation | 1 | 471 | 472 | 2 |
| Pyruvate | 25 | 1,739 | 1,764 | 22 |
| S-archaeol | 1 | 11 | 12 | 1 |
| S-carbamoylation | 1 | 5 | 6 | 2 |
| S-Cyanation | 1 | 5 | 6 | 2 |
| S-cysteinylation | 2 | 13 | 15 | 3 |
| S-diacylglycerol | 58 | 2,137 | 2,195 | 50 |
| Serotonylation | 9 | 44 | 53 | 3 |
| S-linked Glycosylation | 5 | 6 | 11 | 7 |
| S-nitrosylation | 4,172 | 483 | 4,655 | 191 |
| S-palmitoylation | 6,505 | 3,409 | 9,914 | 572 |
| S-selanylation | 0 | 4 | 4 | 0 |
| Stearoylation | 3 | 10 | 13 | 3 |
| Succinylation | 17,973 | 6,387 | 24,360 | 60 |
| Sulfation | 252 | 903 | 1,155 | 129 |
| Sulfhydration | 8 | 169 | 177 | 8 |
| Sulfoxidation | 7,581 | 0 | 7,581 | 124 |
| Sumoylation | 5,889 | 5,731 | 11,620 | 221 |
| Thiocarboxylation | 13 | 82 | 95 | 14 |
| Ubiquitination | 348,308 | 108,349 | 456,657 | 669 |
| UMPylation | 10 | 37 | 47 | 9 |
| 2-Hydroxyisobutyrylation | 0 | 32,392 | 32,392 | 0 |
| **Total in dbPTM** | **2,235,664** | **542,107** | **2,777,771** | **82,444** |

**Table S4. The sources and the data statistics of upstream regulatory proteins.**

| **Source** | **Type of upstream proteins** | **Number of sites or interactions** |
| --- | --- | --- |
| GPS 5.0 | Kinase | 13717 |
| PhosphoELM | Kinase | 2773 |
| PhosphoSitePlus | Kinase | 11388 |
| UniProtKB/Swiss-Prot | Kinase | 13543 |
| UbiNet 2.0 | E3 ligase | 3332 |
| Total in dbPTM 2022 | | 44753 |

**Table S5. Summarized table of all integrated tools and databases associated with PTM analyses**

| **PTM type** | **Number of Integrated tools** | **Tool name** | **Number of integrated databases** | **Database name** |
| --- | --- | --- | --- | --- |
| Acetylation | 1 | DeepAcet | 0 |  |
| ADP-ribosylation | 0 | - | 1 | ADPriboDB 2.0 |
| Arginine methylation | 1 | DeepRMethylSite | 0 | - |
| Carbonylation | 3 | MDD-carb | 1 | CarbonylDB |
|  |  | iCar-PseCp |  |  |
|  |  | iCarPS |  |  |
| Crotonylation | 3 | CKSAAP_CrotSite | 0 | - |
|  |  | iCrotoK-PseAAC |  |  |
|  |  | An unnamed classifier |  |  |
| Cysteine Prenylation | 1 | iPreny-PseAAC | 0 | - |
| Glutarylation | 4 | MDDGlutar | 0 | - |
|  |  | iGlu-Lys |  |  |
|  |  | RF-GlutarySite |  |  |
|  |  | PUL-GLU |  |  |
| Glycation | 2 | GlyStruct | 0 | - |
|  |  | PredGly |  |  |
| Glycosylation | 7 | EnsembleGly | 7 | GlycoEpitope |
|  |  | GlycoMine |  | GlycomeDB |
|  |  | GlycoPP |  | UnicarbKB |
|  |  | GPP |  | GLYCOSCIENCES.de |
|  |  | GS-align |  | GlycoSuiteDB |
|  |  | GlycoEP |  | CFG |
|  |  | iGlycoS-PseAAC |  | ProGlycProt |
| GPI-lipid anchor modified | 4 | big-Pi plant | 0 | - |
|  |  | FragAnchor |  |  |
|  |  | GPI-SOM |  |  |
|  |  | PredGPI |  |  |
| Hydroxylation | 5 | HydLoc | 0 | - |
|  |  | iHyd-LysSite (EPSV) |  |  |
|  |  | iHyd-PseCp |  |  |
|  |  | iHyd-PseAAC |  |  |
|  |  | iHyd-PseAAC (EPSV) |  |  |
| Lipoylation | 2 | LipoPred | 0 | - |
|  |  | LipoSVM |  |  |
| Lysine acetylation | 6 | LysAcet | 1 | CPLA |
|  |  | ASEB |  |  |
|  |  | BRABSB-PHKA |  |  |
|  |  | PAIL |  |  |
|  |  | LAceP |  |  |
|  |  | PSKAcePred |  |  |
| Lysine methylation | 1 | iMethylK_pseAAC | 0 | - |
| Malonylation | 8 | Mal-Light | 0 | - |
|  |  | kmal-sp |  |  |
|  |  | SEMal |  |  |
|  |  | LEMP |  |  |
|  |  | Mal-Prec |  |  |
|  |  | RF-MaloSite and DL-Malosite |  |  |
|  |  | Kmalo |  |  |
|  |  | MaloPred |  |  |
| Meta | 24 | CarSPred | 24 | CPLM |
|  |  | ISSPred |  | CrosstalkDB |
|  |  | ModPred |  | dbPTM |
|  |  | Motifs tree |  | HIstome |
|  |  | NetChop |  | novPTMenzy |
|  |  | NetCorona |  | ProteomeScout |
|  |  | NetPicoRNA |  | PSP |
|  |  | PAProC |  | PTMCode |
|  |  | Pcleavage |  | PTMfunc |
|  |  | PEIMAN |  | PTM-SD |
|  |  | PeptideMap |  | RedoxDB |
|  |  | PHOXTRACK |  | RESID |
|  |  | ProP |  | SysPTM |
|  |  | PTM-X |  | topPTM |
|  |  | PyTMs |  | VPTMdb |
|  |  | MusiteDeep |  | PRISMOID |
|  |  | MUscADEL |  | iProteinDB |
|  |  | PTM-ssMP |  | AWESOME |
|  |  | An unnamed classifier |  | YAAM |
|  |  | PTMscape |  | ActiveDriverDB |
|  |  | MultiLyGAN |  | PTMsnp |
|  |  | Plant PTM Viewer |  | FAT-PTM |
|  |  | PTM-Logo |  | neXtProt |
|  |  | RESTful API |  | iPTMnet |
| Methylation | 4 | BPB-PPMS | 1 | PubMeth |
|  |  | iMethyl-PseAAC |  |  |
|  |  | MASA |  |  |
|  |  | MeMo |  |  |
| Multiple cysteine modifications | 1 | pCysMod | 0 | - |
| N-acetylation | 2 | NetAcet | 0 | - |
|  |  | N-Ace |  |  |
| Myristoylation | 1 | NMT | 1 | MYRbase |
| N-Glycosylation | 3 | NetNGlyc | 4 | GlycoFish  GlycoFly  GlycoProtDB  UniPep |
|  |  | GECS |  |  |
|  |  | N-GlycoGo |  |  |
| Neddylation | 1 | NeddPred | 0 | - |
| Nitration | 2 | NTyroSite | 0 | - |
|  |  | PredNTS |  |  |
| O-GlcNAcylation | 2 | YinOYang | 2 | dbOGAP |
|  |  | DictyOGlyc |  | O-GlcNAcAtlas |
| O-Glycosylation | 2 | NetOGlyc | 2 | O-GlycBase |
|  |  | Oglyc |  | OGP |
| Phosphoglycerylation | 7 | PhoglyStruct | 0 | - |
|  |  | Bigram-PGK |  |  |
|  |  | iDPGK |  |  |
|  |  | RAM-PGK |  |  |
|  |  | iPGK-PseAAC |  |  |
|  |  | EvolStruct-Phogly |  |  |
|  |  | predPhogly-Site |  |  |
| Phosphorylation | 39 | AMS | 22 | dbPPT  dbPSP  HPRD  LymPHOS  MAPRes  P3DB  PepCyber:P~PEP  PHOSIDA  PhosPhAt  Phospho.ELM  Phospho3D  PhosphoGRID  PhosphoNET  PhosphoPOINT  PhospoPep  PhosSNP  SubPhos  dbPSP 2.0  EPSD  qPhos  dbPAF  Scop3P |
|  |  | CKSAAP_PhSite |  |  |
|  |  | CRPhos |  |  |
|  |  | DAPPLE |  |  |
|  |  | DISPHOS |  |  |
|  |  | GPS |  |  |
|  |  | HMMpTM |  |  |
|  |  | KinasePhos |  |  |
|  |  | KinomeXplorer |  |  |
|  |  | MetaPredPS |  |  |
|  |  | Musite |  |  |
|  |  | NetPhorest |  |  |
|  |  | NetPhos |  |  |
|  |  | NetPhosK |  |  |
|  |  | NetPhosYeast |  |  |
|  |  | phos_pred |  |  |
|  |  | Phos3D |  |  |
|  |  | PhoScan |  |  |
|  |  | PHOSFER |  |  |
|  |  | PHOSITE |  |  |
|  |  | PhosphoPICK |  |  |
|  |  | PhosphoRice |  |  |
|  |  | PhosphoSVM |  |  |
|  |  | pkaPS |  |  |
|  |  | PKIS |  |  |
|  |  | PlantPhos |  |  |
|  |  | PostMod |  |  |
|  |  | PPRED |  |  |
|  |  | PPSP |  |  |
|  |  | Predikin |  |  |
|  |  | PredPhospho |  |  |
|  |  | PSEA |  |  |
|  |  | PTMPred |  |  |
|  |  | RLIMS-P |  |  |
|  |  | ViralPhos |  |  |
|  |  | iPhosY-PseAAC |  |  |
|  |  | iPhosT-PseAAC |  |  |
|  |  | iPhosH-PseAAC |  |  |
|  |  | PhosphOrtholog |  |  |
| Prenylation | 2 | PrePS | 1 | PRENbase |
|  |  | SPrenylC-PseAAC |  |  |
| Propionylation | 2 | PropPred |  |  |
|  |  | An unnamed classifier |  |  |
| Protein cysteine modifications | 0 | - | 1 | iCysMod |
| Pupylation | 3 | GPS-PUP | 1 | PupDB |
|  |  | PUP-Fuse |  |  |
|  |  | PupStruct |  |  |
| S-glutathionylation | 2 | GSTPred | 1 | dbGSH |
|  |  | DeepGSH |  |  |
| S-nitrosylation | 4 | GPS-SNO | 1 | dbSNO |
|  |  | iSNO-AAPair |  |  |
|  |  | iSNO-PseAAC |  |  |
|  |  | PSNO |  |  |
| S-Palmitoylation | 2 | MDD-Palm | 0 | - |
|  |  | SPalmitoylC-PseAAC |  |  |
| S-sulfenylation | 2 | SVM-SulfoSite | 0 | - |
|  |  | iSulf-Cys |  |  |
| S-sulphenylation | 2 | DeepCSO | 0 | - |
|  |  | SIMLIN |  |  |
| Succinylation | 12 | iSuc-PseAAC | 0 | - |
|  |  | SSKM_Succ |  |  |
|  |  | HybridSucc |  |  |
|  |  | CNN-SuccSite |  |  |
|  |  | SuccSite |  |  |
|  |  | Inspector |  |  |
|  |  | Success |  |  |
|  |  | GPSuc |  |  |
|  |  | PSuccE |  |  |
|  |  | IFS-LightGBM |  |  |
|  |  | DeepSuccinylSite |  |  |
|  |  | SuccinSite |  |  |
| SUMOylation | 7 | GPS-SUMO | 0 | - |
|  |  | JASSA |  |  |
|  |  | SUMOhydro |  |  |
|  |  | SUMOplot |  |  |
|  |  | SUMOgo |  |  |
|  |  | HseSUMO |  |  |
|  |  | SumSec |  |  |
| Tyrosine nitration | 1 | pNitro-Tyr-PseAAC | 0 | - |
| Tyrosine sulfation | 2 | The Sulfinator | 0 | - |
|  |  | iSulfoTyr-PseAAC |  |  |
| Ubiquitination | 7 | hCKSAAP_UbSite | 2 | UbiProt  UbiNet 2.0 |
|  |  | iUbiq-Lys |  |  |
|  |  | UbiPred |  |  |
|  |  | UbiProber |  |  |
|  |  | UbPred |  |  |
|  |  | DeepUbi |  |  |
|  |  | An unnamed classifier |  |  |
| 2-Hydroxyisobutyrylation | 1 | DeepKhib | 0 | - |
